# Flt-1 in colorectal cancer cells is required for the tumor invasive effect of placental growth factor through a p38-MMP9 pathway

**DOI:** 10.1186/1423-0127-20-39

**Published:** 2013-06-21

**Authors:** Shu-Chen Wei, Po-Nien Tsao, Meng-Tzu Weng, Zhifang Cao, Jau-Min Wong

**Affiliations:** 1 Departments of Internal Medicine, National Taiwan University Hospital and College of Medicine, Taipei 100, Taiwan; 2 Department of Pediatrics, National Taiwan University Hospital and College of Medicine, Taipei 100, Taiwan; 3 Department of Internal Medicine, Far Eastern Memorial Hospital, Taipei 100, Taiwan; 4 Gastrointestinal Unit, Massachusetts General Hospital, Boston, MA 02114, USA

**Keywords:** Colorectal cancer, Flt-1, PlGF, Invasion, Migration, MMP9

## Abstract

**Background:**

Placenta growth factor (PlGF), a dimeric glycoprotein with 53% homology to VEGF, binds to VEGF receptor-1 (Flt-1), but not to VEGF receptor-2 (Flk-1), and may function by modulating VEGF activity. We previously have showed that PlGF displays prognostic value in colorectal cancer (CRC) but the mechanism remains elucidated.

**Results:**

Overexpression of PlGF increased the invasive/migration ability and decreased apoptosis in CRC cells showing Flt-1 expression. Increased migration was associated with increasing MMP9 via p38 MAPK activation. Tumors grew faster, larger; with higher vascularity from PlGF over-expression cells in xenograft assay. In two independent human CRC tissue cohorts, PlGF, MMP9, and Flt-1 expressions were higher in the advanced than the localized disease group. PlGF expression correlated with MMP9, and Flt-1 expression. CRC patients with high PlGF and high Flt-1 expression in tissue had poor prognosis.

**Conclusion:**

PlGF/Flt-1 signaling plays an important role in CRC progression, blocking PlGF/Flt-1 signaling maybe an alternative therapy for CRC.

## Background

Colorectal cancer (CRC) is the second leading cause of death from cancer in Western countries [1] and the third most common cancer in Taiwan [2]. The fact that tumor growth and metastasis rely on angiogenesis has been widely accepted [3]. Increased angiogenesis in the primary tumor of CRC has been associated with poor prognosis and relapse of disease [4,5]. Previously, we have demonstrated that PlGF expression was up-regulated in CRC tissue. Immunohistochemical staining analysis showed that PlGF was expressed mainly in tumor cells and Flt-1 was expressed in tumor cells as well as in endothelial cells. The extent of up-regulation correlated with disease progression and patient survival [2]. We further demonstrated that the preoperative serum placental growth factor levels were higher in CRC patients and could be used as a prognostic indicator for recurrence and survival of CRC [6]. However, the underlie mechanism of PlGF and its receptor (Flt-1) regulating the CRC carcinogenesis remains unknown.

Placental growth factor (PlGF), a dimeric glycoprotein with 53% homology to VEGF [7,8] binds to VEGF receptor-1 (Flt-1), but not to VEGF receptor-2 (Flk-1), and may function by modulating VEGF activity [9]. In addition to the angiogenic effect, PlGF expression has been reported in renal cell carcinomas, thyroid and germ-cell tumors [10], as well as the meningiomas [11]. It also has been shown that in human gastric cancer, breast, renal, and lung (non-small cell) cancer, PlGF was over-expressed and displayed prognostic value [12-15].

In contrast to the expression of both Flk-1 and Flt-1 in endothelial cells, Flt-1 is widely expressed in many non-endothelial cell types, including hepatocytes, bone marrow progenitor cells, monocytes, macrophages, neural cells, vascular smooth muscle cells, and various tumor cells [16-19]. Therefore, in addition to its role in angiogenesis, Flt-1 might mediate a variety of hitherto unappreciated biological functions, such as liver regeneration, inflammatory process and cancer metastasis [18,19].

Here we provide evidence that, in addition to the angiogenesis, PlGF/Flt-1 signaling in colorectal cancer cells can promote CRC invasion through a p38-MMP9 pathway and the association with the poor prognosis of CRC patients was validated by two different CRC cohorts.

## Methods

### Ethics statements

This study was approved by the Research Ethics Committee of the National Taiwan University Hospital (201107063RC).

### Cell culture

Human 293 T cells and colon cancer cell lines SW480, HCT116, HT29, and LoVo were obtained from the American Type Culture Collection (ATCC, Manassas, VA). They were cultured in DMEM with 10% fetal bovine serum and 1% Penicillin/Streptomycin. Cells were grown at 37°C in a 5% CO_2_ atmosphere within a humidified incubator.

### Human CRC tissue

Colorectal cryosections were prepared from colorectal cancer surgical samples which were collected from September 2000 to June 2003 after obtaining the written informed consent, following the guidelines set forth by the Research Ethics Committee of the National Taiwan University Hospital. All tissues were freshly frozen or immersed in optimal cutting temperature compound (OCT) (Ames Company, Elkhart, IN), and kept at −80°C until use. Clinical staging of cancers was determined based on the UICC-TNM classification. Stages I and II were collectively termed the localized disease group (n = 47) and stages III and IV as advanced disease group (n = 33).

### Reagents and antibodies

The p38 MAPK inhibitor SB203580 and the MMP9 inhibitor were purchased from Merck KGaA (Darmstadt, Germany), and Zeocin from Invitrogen (Carlsbad, CA). Polyclonal rabbit antibodies against phospho-JNK, phospho-ERK 1/2, phospho-p38, and total JNK, ERK1/2, and p38 were purchased from Cell Signaling Technology (Danvers, MA). Other antibodies used were mouse monoclonal antibodies to FLAG M2 (α-FLAG, Sigma Chemical Co., St. Louis, MO), rat polyclonal anti-CD31 (BD Biosciences, San Jose, CA), rabbit polyclonal anti-PlGF (C-20) (Santa Cruz, CA), anti-Factor VIII (Biomeda Corporation, Foster, CA), anti-cleaved Caspase-3 from Cell Signaling Technology (Danvers, MA) and anti-MMP-9 (Abcam, Cambridge, UK). Recombinant PlGF was purchased from R&D Systems, Inc. (Minneapolis, MN).

### Plasmids, small interfering RNA, and transfection

The human PlGF expression vector was generated by PCR amplification of full length of PlGF cDNA and inserted into the multiple cloning site of pcDNA4/TO-flag-strepII N1 Vector (Flag-StrepII tandem tag at the N-terminus, derived from Invitrogen pcDNATM4/TO vector). HA-tagged human Flt-1 expression vector, pCMV-TAG-Flt-1 (HA-Flt-1), was generated by PCR amplification of Flt-1 cDNA and insertion into the multiple cloning sites of pCMV-TAG vector (Invitrogen, Carlsbad, CA). Negative control siRNA, siFlt-1, sip38 (p38α), and siPlGF were purchased from Ambion Inc. (Austin, TX). LoVo cells were transfected using Lipofectamine 2000 (Invitrogen, Carlsbad, CA). For generating the stable cell lines (LoVo, SW480, HT29 and HCT116), selection with Zeocin (100 μg/ml) began one day after transfection and maintained under same Zeocin condition.

### RNA extraction and quantitative PCR

Total RNA from cell lines and tissue was isolated using an RNA extraction kit (Qiagen Inc., Valencia, CA), according to the manufacturer’s instructions. For reverse transcription, 1 μg of total RNA was transcribed using the iScript cDNA synthesis kit (Bio-Rad, Hercules, CA). Quantitative PCR was performed in a DNA Engine Opticon 2 (Bio-Rad, Hercules, CA) using iQ SYBR Green supermix (Bio-Rad, Hercules, CA) with human GAPDH as an internal control. Primers sequences used for RT-PCR were as the following, PlGF: Forward-5′-TGCGGCGATGAGAATCTGC-3′, Reverse-5′-AGCGAACGTGCTGAGAGAAC-3′; Flt-1: Forward-5′-AGCAGGTGCTTGAAACCGTAG-3′, Reverse-5′-GTCGCAGGTAACCCATCTTTT-3; MMP9: Forward-5′-GGGACGCAGACATCGTCATC-3′, Reverse-5′-TCGTCATCGTCGAAATGGGC-3′.

### Western blot analysis

Cells were lysed with NP-40 lysis buffer and centrifuged at 15,000 rpm for 20 minutes at 4°C. The supernatant was assayed for protein concentration (Bradford). Equal amounts of protein (150-250 μg/lane) was added to Tris-Glycine SDS sample buffer (Invitrogen, Carlsbad, CA) and separated on 4-12% gradient Tris-Glycine gels (Invitrogen, Carlsbad, CA). Following electrophoresis, proteins were electro-transferred to polyvinylidene difluoride membranes and blocked with 5% bovine serum albumin in TBST (Tris-Buffered Saline and Tween 20). Membranes were then incubated with specific primary antibody overnight, washed, then incubated with appropriate secondary antibody conjugated to horseradish peroxidase, and developed using ECL (PerkinElmer Life Sciences). Membranes were stripped and re-probed with anti-total MAPK (for MAPK antibodies) or anti-actin to confirm equal protein loading.

### Immunohistochemistry

Frozen sections (8 μm thick) were stained by using the NoVo Link Polymer Detection System (Leica, Biosystems Newcastle Ltd, UK), followed by AEC substrate kit (Vector Laboratories Inc. Burlingame, CA), according to the manufacturer’s instruction. Tissues were counterstained with Mayer’s hematoxylin. Isotype antibody was used as the staining negative control.

### ELISA (enzyme linked immunoabsorbent assay)

Concentrations of PlGF in cell culture medium were quantified using a Quantikine PlGF immunoassay (R&D Systems, Inc., Minneapolis, MN), as previously described [2]. Concentrations of PlGF were expressed as pg/ml of protein.

### Apoptosis assay

Apoptosis was quantified by staining with the Annexin V-FITC kit (Strong Biotech Corporation, Taipei, Taiwan), data collection by flow cytometer (Becton Dickinson) and analyzed by FlowJo 7.2 software. For tissue sections, immunohistochemical staining with cleaved caspase 3 antibody was used for apoptosis analysis.

### *In vitro* invasion and migration assay

The invasive activity of the cancer cells was examined using a membrane invasion culture system in which a polycarbonate membrane with 8-μm pores (Millipore., Billerica, MA) coated with Matrigel (R&D Systems, Inc., Minneapolis, MN) at 5 mg/mL was placed between the upper and lower wells of a membrane invasion culture system chamber. 5 × 10^4^ cells were placed into each upper well of the chamber. After incubating for 48 hours at 37°C, cells that had migrated through the coated membrane were removed from the lower chamber with 1 m*M* EDTA in PBS and dot blotted onto a polycarbonate membrane with 3-μm pores. Blotted cells were stained with Giemsa (Sigma Chemical Co., St. Louis, MO), and the number of cells on each blot was counted under a microscope at a magnification of × 50. Each experiment was performed three times, and each sample was assayed in triplicate. The migration assay was also performed using the same procedure, except without the Matrigel coating.

### Proliferation assay

Cell growth was measured using MTS [3-(4, 5-dimethylthiazol-2-yl)-5-(3-carboxymethoxyphenyl)-2-(4-sulfophenyl)-2H-tetrazolium, inner salt] in the form of the CellTiter 96 Aqueous One Solution Cell Proliferation Assay kit (Promega, UK), according to the manufacturer’s instructions. Briefly, 1,500 cells/well were plated in 96 well plates and allowed to proliferate for two days. The OD 490 correlated with cell density. All assays were repeated thrice.

### Tumor xenograft growth assay

LoVo PlGF expression and mock (vector only) stable cells in log phase were trypsinized and washed twice with 137 mM NaCl, 5 mM KCl, 4 mM NaHCO3, 0.5 mM EDTA, 0.1% (w/v) glucose. 10^6^ cells in 100 μl PBS were injected subcutaneously into the backs of 8-week-old SCID mice. The body weight and tumor size were recorded twice per week. After 14 weeks, the mice were sacrificed. Tumor size was measured and further studies were performed on the tissue.

### Microvessel density measurement

Using light microscopy at 200X magnification, the vascular counts were measured for the tissue section staining with CD31. The three areas with the highest number of discrete microvessels were chosen for analysis and the region with the highest microvessel counts was selected as the final result for that case [20]. Any immunoreactive endothelial cells that were separate from adjacent microvessels were considered to be countable vessels.

### Gene expression dataset from the Gene Expression Omnibus (GEO) database analysis

The colorectal cancer patient gene expression data was available on Gene Expression Omnibus (GEO) with accession number GSE17536. Expression data were analyzed using GeneSpring GX software (Agilent Technologies). High and low PlGF/Flt-1 expression was defined according to the median expression level in each group.

### Statistical analysis

Statistical differences between groups were analyzed by Student’s *t* test or Mann–Whitney U test. Data was expressed as means ± standard errors (SE). Correlations between PlGF and MMP-9 expression levels were analyzed by Spearman’s correlation coefficient. A *p* value of 0.05 was considered to be statistically significant. The body weight difference along with the time between the LoVo-PlGF and control group, in terms of group effect, time effect, and their interactive effect, were analyzed using the mixed model. Based on fit statistics for Akaike information criterion (AIC) and Bayesian information criterion (BIC) criteria, the repeated measures were modeled using the first-order ante dependence for the covariance structure [21].

## Results

### Expression of PlGF and its receptor Flt-1 in CRC cell lines

**Figure 1:**
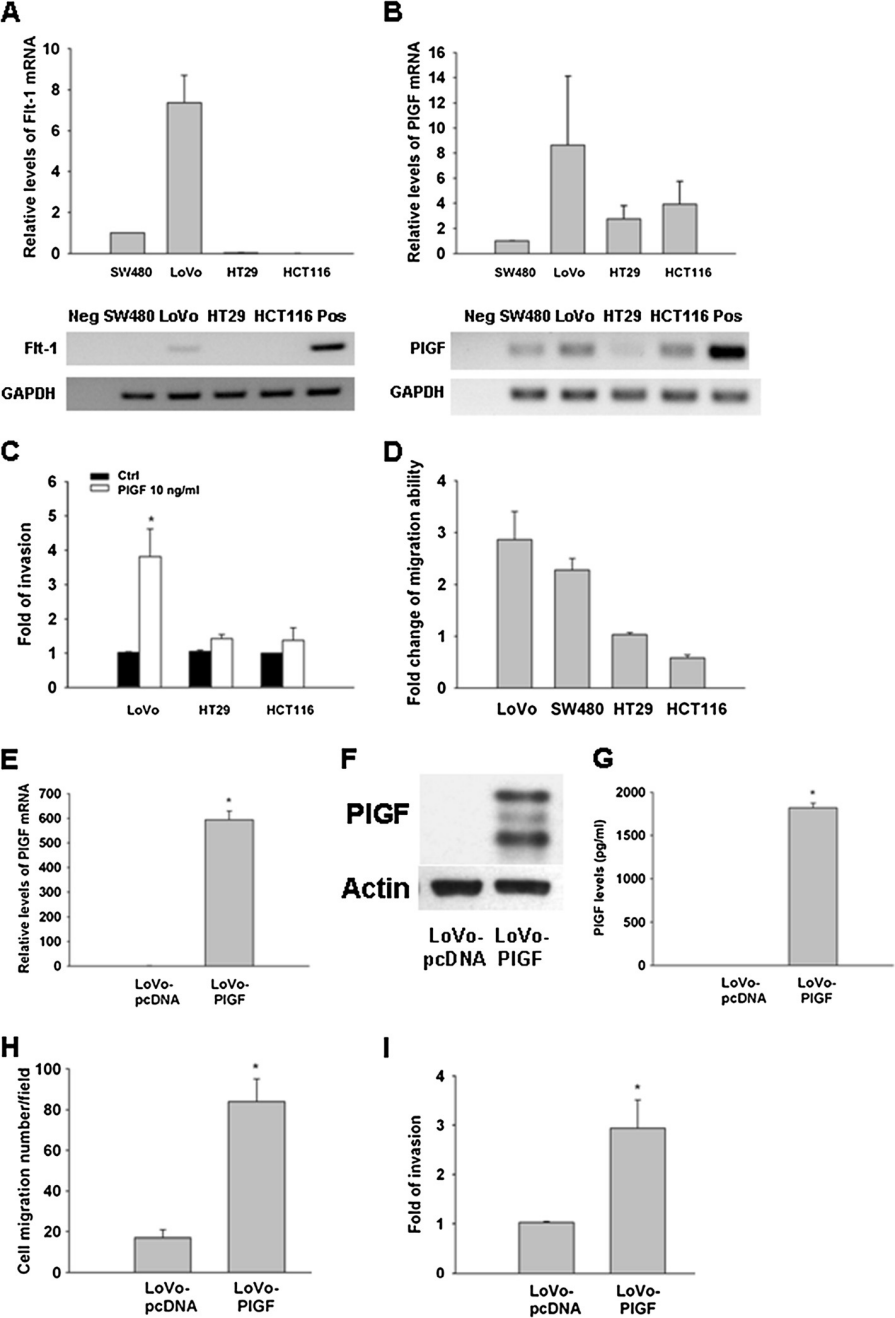
**Expression and biological function of PlGF and Flt-1 in CRC cell lines.** Quantitative PCR levels of Flt-1 (**A**) and PlGF (**B**) in CRC cell lines. The expression of SW480 was arbitrarily defined as the reference for comparison. (**C**) PlGF recombinant protein (10 ng/ml) increased the invasion ability in LoVo but not HT29 and HCT116 cells; (**D**) stable overexpression of PlGF increased the migration ability in LoVo and SW480 cells, but not in HT29 and HCT116 cells. PlGF level in the LoVo-PlGF (PlGF overexpressioin) cells was validated by quantitative RT-PCR (**E**), Western blot (**F**) (anti-PlGF at 200 ng/ml) and ELISA (**G**). The migration (**H**) and invasion (**I**) abilities increased in LoVo-PlGF cells as compared to LoVo-pcDNA, respectively. (Neg: negative control; Pos; positive control. * indicated as P < 0.05).

When we arbitrarily used SW480 expression as 1, Flt-1 expression in LoVo cells was 7.4, and 5971 for the Flt-1 overexpression in 293 T cells; Flt-1 was not detectable in the negative control and barely detectable in both HT29 and HCT116 cell lines (Figure 1A). In contrast, PlGF was expressed (within folds of change) in the four CRC cell lines (Figure 1B) by quantitative PCR.

### Flt-1 is required for PlGF-induced invasive/migration ability of CRC cells exogenously added PlGF or overexpression of PlGF increased the invasive/migration ability of CRC cells expressing Flt-1

Exogenous PlGF significantly increased the invasive ability of LoVo cells, which expressed the highest Flt-1 levels of the cell lines tested by up to four fold. Conversely, HT29 and HCT116, cell lines in which Flt-1 was virtually undetectable, did not respond to exogenously added PlGF (Figure 1C). To further confirm the role of PlGF in CRC cancer cells, we generated the PlGF-over-expression stable clones in LoVo, SW480, HT29 and HCT116 cells as well as their empty vector control cells. The over-expression of PlGF in these stable clones have been validated and monitored periodically by quantitative PCR, which we got hundreds of fold increased expression of PlGF than the control cell lines. Migration assay was performed to compare the stable clones with and without PlGF and normalized to the empty vector control. Migration ability increased in LoVo and SW480 with stable expression of PlGF, but no change in HT29 cells and even decreased in HCT116 cells (Figure 1D). This data suggests that Flt-1 receptor may be critical for PlGF induced tumor cell invasion in CRC cells.

#### Overexpression of PlGF in CRC cells mildly decreased apoptosis, but did not affect their proliferative status

Stable clones have been validated for the presence of the transgene and for overexpression of PlGF protein by quantitative RT-PCR, Western blot and ELISA (Figure 1E, F and G). According to the datasheet, it showed PlGF (C-20) can be used for detecting variants of PlGF of human origin. The overexpressed PlGF was double confirmed by immunoblotting with Flag and PlGF. As increased invasion/migration was shown most prominently with the LoVo stable clone for PlGF, LoVo-PlGF and its control LoVo-pcDNA were used for the following experiments. The migration and invasion abilities increased 4.9 and 3.9 fold in LoVo-PlGF cells as compared to LoVo-pcDNA, respectively (Figure 1H, I). There was minor apoptosis difference by PI and annexin V staining between LoVo-pcDNA and LoVo-PlGF cells, 3.2% and 1.3%, respectively, and no difference in the proliferation status by MTS assay between these two cell lines (data not shown).

#### The increased migration ability of LoVo-PlGF cells is through increasing MMP9 expression via p38 MAPK activation

**Figure 2:**
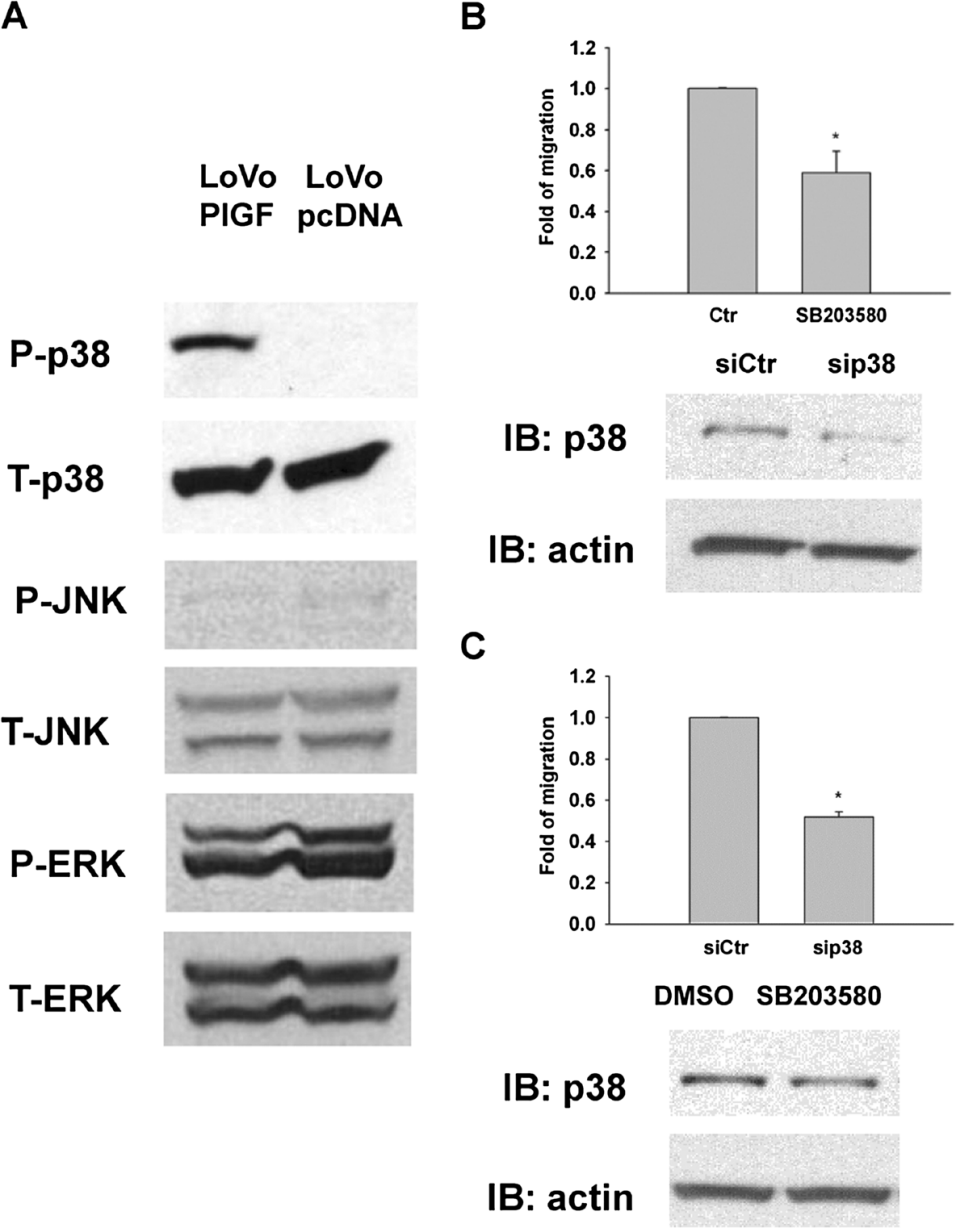
**Increased migration of LoVo-PlGF cells was due to increasing p38 MAPK activation.** (**A**) Phosphorylation levels of p38, JNK and ERK are shown along with total protein levels of p38, JNK and ERK in LoVo-PlGF and LoVo pcDNA control cells. (**B**) Inhibition of p38 by chemical inhibitor, SB203580 (20 μM) as well as by siRNA of p38 (30 pmole/ml) (**C**), decreased the migratory ability of LoVo-PlGF. * indicated as P < 0.05.

In LoVo cells with overexpression of PlGF, we found that phosphorylation of p38 increased but there was no increase in the phosphorylation levels of ERK and JNK MAP kinases (Figure 2A). It might be due to the exposure time which showing the phospho-ERK at both pcDNA and PlGF group. From the results, we could say there was no significant difference between the pcDNA and PlGF group for the expression of ERK (both phospho and total form). Inhibition of p38, either by using p38 chemical inhibitors (Figure 2B) or siRNA (Figure 2C), decreased the ability of LoVo-PlGF cells to migrate. All experiments had been performed at least 3 times.

**Figure 3:**
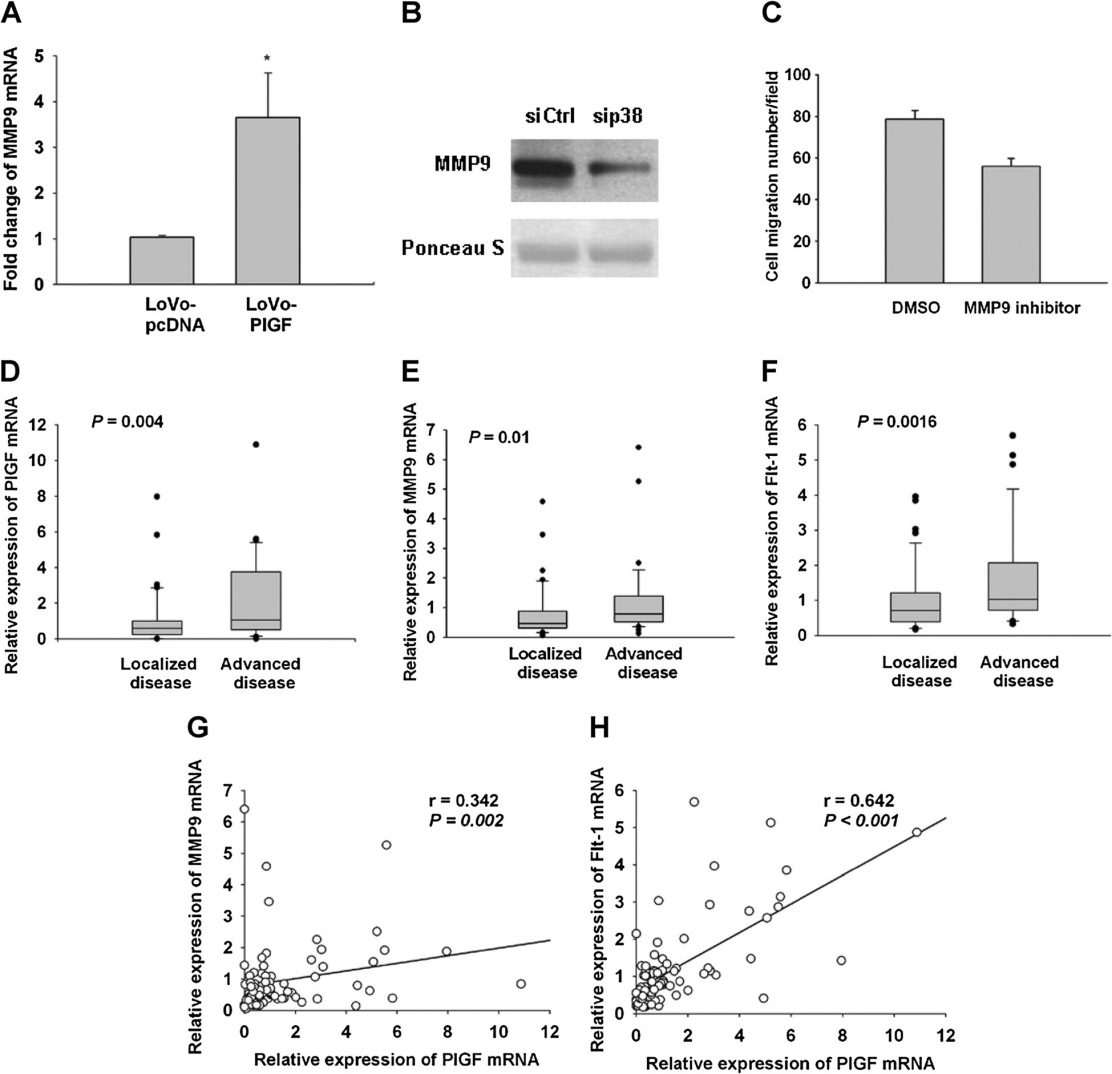
**PlGF induced MMP9 expression via p38 MAPK activation.** (**A**) MMP9 expression was significantly increased in LoVo-PlGF cells compared to LoVo-pcDNA cells as assessed by quantitative PCR. (**B**) Inhibition of p38 by siRNA (30 pmole/ml) decreased the expression of MMP9 in LoVo-PlGF cells as detected by Western blotting. (**C**) By using the chemical inhibitor of MMP9 (30 μM), the migration ability was inhibited in LoVo-PlGF cells. In human colorectal cancer tissue, PlGF (**D**), MMP9 (**E**) and Flt-1 (**F**) expression were significantly higher in the advanced than localized disease group. There was a strongly positive correlation between the expression of PlGF and MMP9 (**G**), as well as PlGF and Flt-1 (**H**). * indicated as P < 0.05.

Several lines of evidence have shown that p38 activation is required for MMP9 expression, which has been linked to tumor migration and invasion [22,23]. We therefore checked MMP9 expression in LoVo-PlGF cells. Indeed, MMP9 expression was significantly increased in LoVo-PlGF cells compared to control at the message level (Figure 3A), and inhibition of p38 by both siRNA and chemical inhibition both decreased the expression of MMP9 at the protein level (Figure 3B and data not shown). The migration ability was inhibited by using the chemical inhibitor of MMP9 (Figure 3C).

To investigate the clinical significance of this *in vitro* finding, we checked PlGF, Flt-1, and MMP9 expression in 80 human colorectal cancer tissues at the message level. We found, indeed, PlGF, MMP9, and Flt-1 expression were significantly higher in the advanced CRC group than the localized CRC group (Figure 3D, E and F). Moreover, the expression of MMP9 in human CRC tumor samples significantly correlated with the PlGF expression levels in the samples (Figure 3G), as well as the PlGF expression and Flt-1 expression (Figure 3H).

#### Flt-1 is required for PlGF-induced p38 phosphorylation and its results of promoting CRC cells migration/invasion

**Figure 4:**
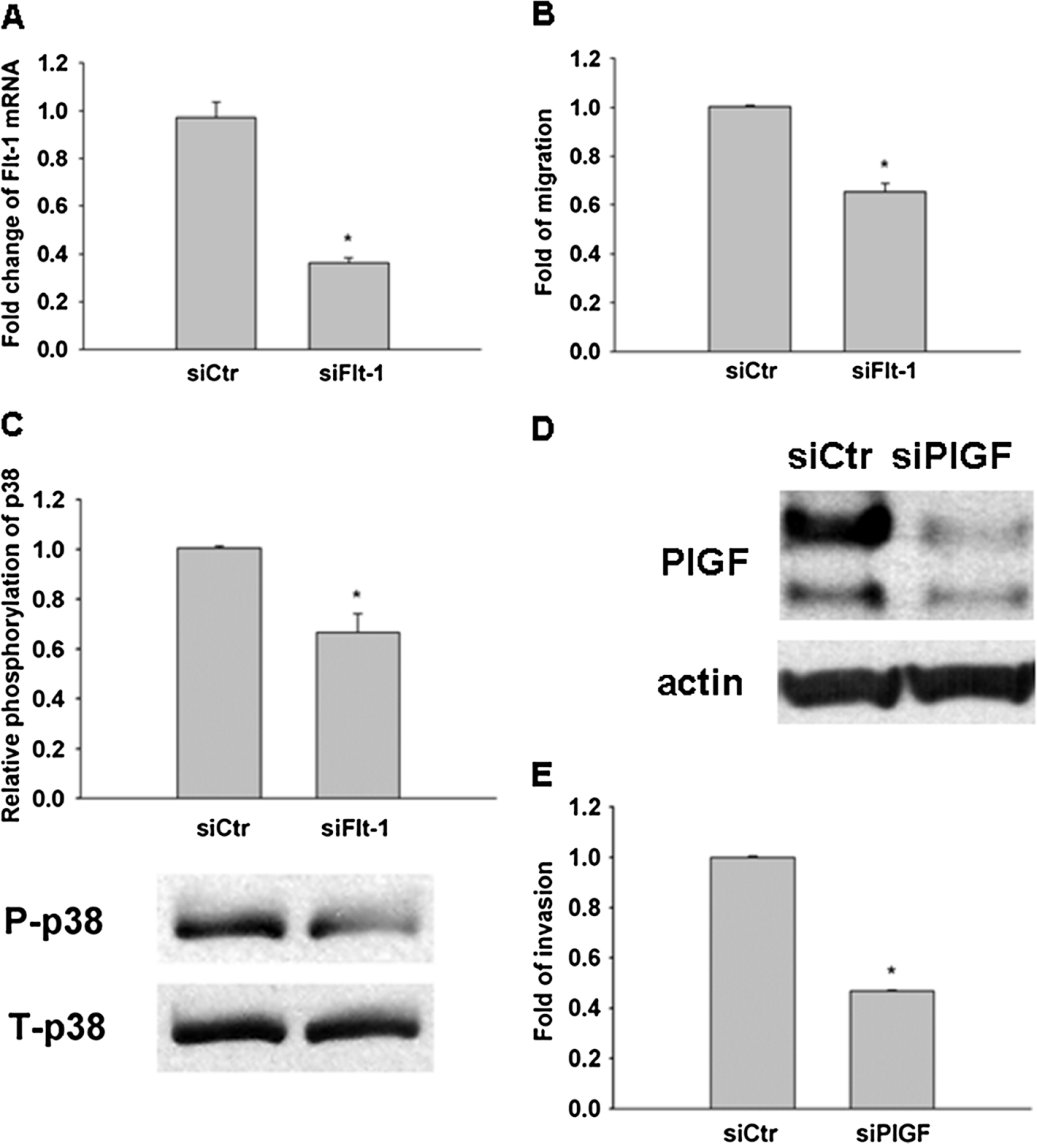
**Flt-1 is required for PlGF-induced p38 phosphorylation, PlGF and Flt-1 were both important in promoting CRC cancer cell migration/invasion.** siRNA (30 pmole/ml) inhibited Flt-1 expression and migratory ability in LoVo-PlGF cells as monitored by quantitative PCR (**A**) and migration assay (**B**). (**C**) Knockdown Flt-1 significantly attenuated p38 phopsphorylation in LoVo-PlGF. (**D**, **E**). The invasion ability of CRC cells decreased when PlGF was inhibited by siRNA. * indicated as P < 0.05.

Next, we asked whether Flt-1 expression is required for PlGF-induced tumor invasion since only the Flt-1 expressing CRC cell line responded to exogenous PlGF. To address this question we used a siRNA approach to inhibit Flt-1 expression in LoVo-PlGF cells (Figure 4A). We have tried the Western blot for Flt-1, however, we could only detect the over-expressed one but not the endogenous one. This may be due to the low expression level of endogenous Flt-1. Therefore, we could only show the data by quantitative PCR. The migration ability decreased when Flt-1 levels were reduced by siRNA (Figure 4B). This data indicates Flt-1 is required for PlGF-induced CRC cell migration. To further confirm this result, we checked the effect of siFlt-1 on p38 phosphorylation in LoVo-PlGF cells. Indeed, downregulation of Flt-1 significantly attenuated the phosphorylation of p38 in LoVo-PlGF cells (Figure 4C). We also found that both migration and invasion ability decreased in LoVo-PlGF cells when the PlGF was knocked down (validated in Figure 4D) by using the siRNA approach (Figure 4E).

#### Tumor progression was enhanced in LoVo-PlGF cells *ex vivo*

**Figure 5:**
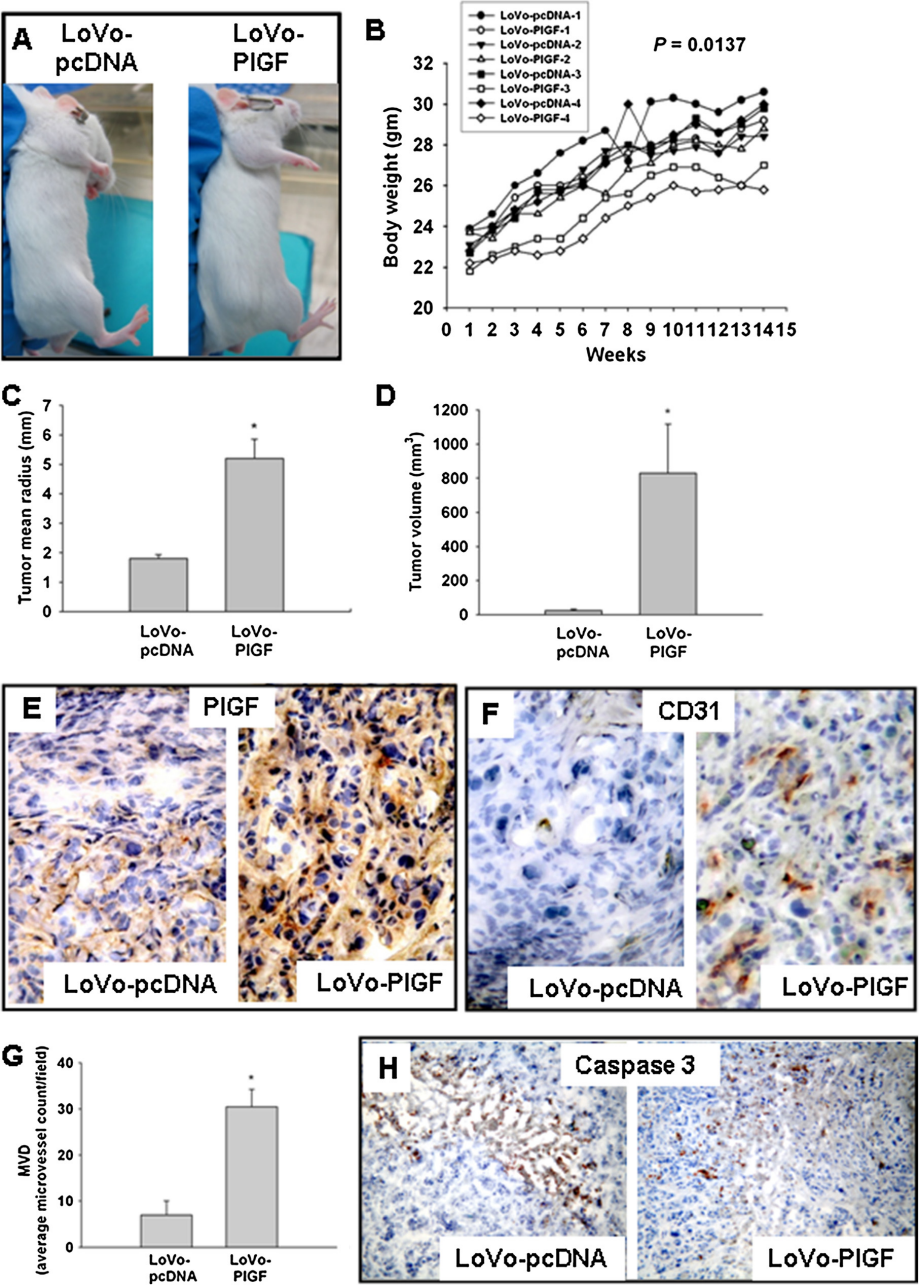
**Tumors grew faster and larger in animals receiving LoVo-PlGF xenografts.** (**A**) At the 10th week after injection, tumors were visible in the LoVo-PlGF group but not in the LoVo-pcDNA group. (**B**) The LoVo-PlGF group lagged behind in body weight gain. Both the tumor radius (**C**) and tumor volumes (**D**) were larger in the LoVo-PlGF group than the control group. (**E**) PlGF expression was significantly higher in the tumor tissue induced by LoVo-PlGF cells. (**F**) Angiogenesis was also more advanced in the LoVo-PlGF group, with the microvessel density shown in (**G**). (**H**) Less apoptosis in LoVo-PlGF group than the control group. * indicated as P < 0.05.

To confirm the role of PlGF in CRC *ex vivo*, tumor xenograft assays were performed. During the observation period, three of the four LoVo-PlGF cells implanted mice had palpable nodules as early as the 3rd week, and all of them had measurable tumors by the end of the 14 week experimental period. In contrast, only two of the four mice in the LoVo-pcDNA group had palpable nodules, detectable only in the 10th week (Figure 5A). The LoVo-PlGF group also gained weight slower than the control group (Figure 5B, group effect, P = 0.0137). The body weight difference became even more significant during follow-up (interactive effect between time and group; P < 0.0001). Mice were sacrificed following week 14. Both the tumor radius and tumor volumes were larger in the LoVo-PlGF group (Figure 5C and D). PlGF expression was indeed significantly higher in the tumor tissue induced by LoVo-PlGF cell implantation (Figure 5E). LoVo-PlGF induced tumors had higher vascularity (Figure 5F), higher microvessel density (Figure 5G), and less caspase 3 staining (Figure 5H) than the control group.

##### High expression of PlGF and Flt-1 in CRC tissues predicts worse prognosis

**Figure 6:**
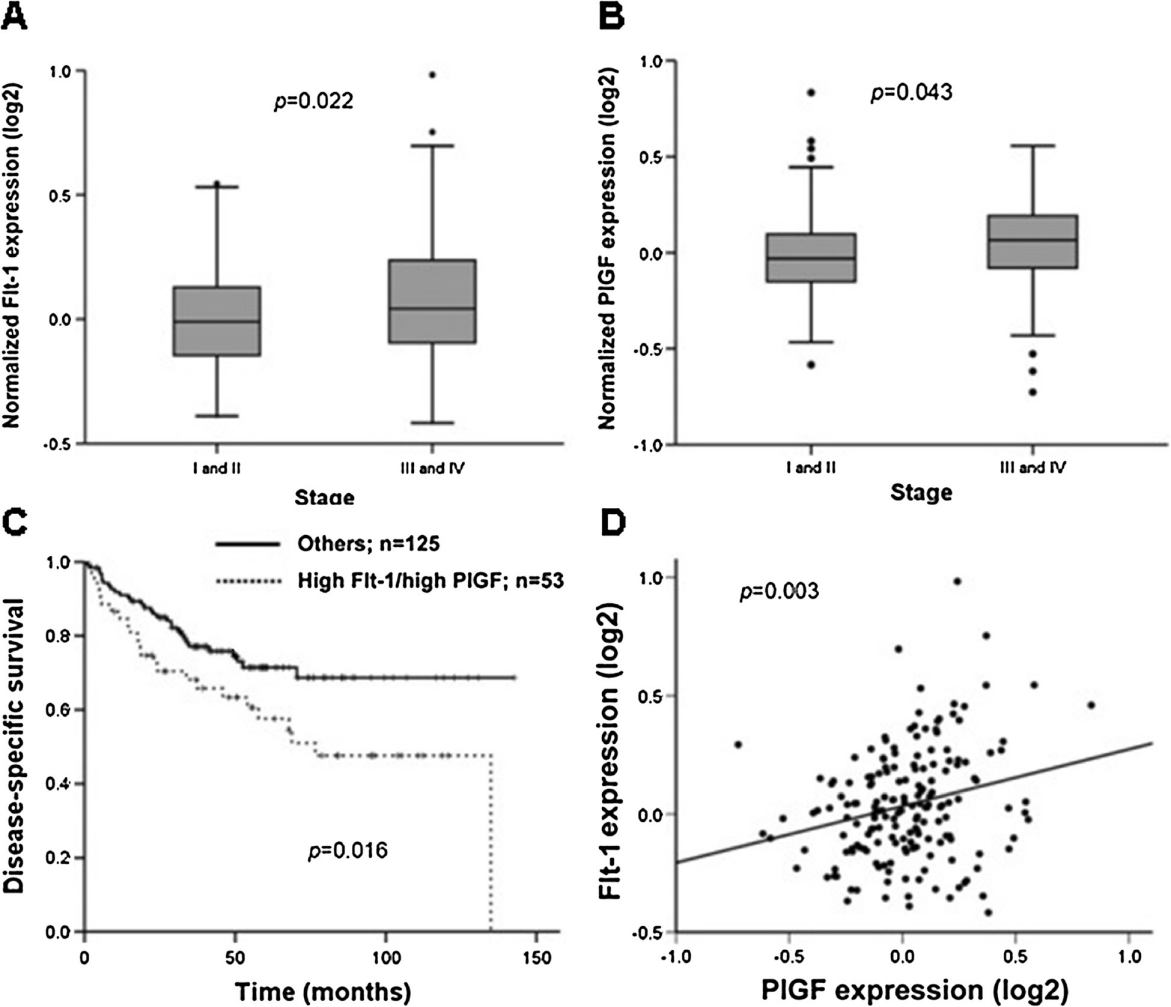
**PlGF correlated with Flt-1 expression in CRC tissue and patients with higher expression of PlGF and Flt-1 had worse prognosis in another CRC cohort.** (**A, B**) higher Flt-1 and PlGF mRNA expressions were observed in stage III-IV disease (advanced disease) compared to stage I-II disease (localized disease). (**C**) Patients with high Flt-1 and high PlGF expression had shorter survival. (**D**) Flt-1 expression correlated well with PlGF levels.

We further analyzed a publicly available gene expression dataset from the GEO database. In this cohort, higher PlGF and Flt-1 mRNA expressions were observed in stage III-IV diseases (advanced disease) compared to stage I-II disease (localized disease) (Figure 6A and B). Patients that had high Flt-1 and high PlGF expression had shorter survival (*p* = 0.016) (Figure 6C). Flt-1 expression was correlated with PlGF (*p* = 0.003, correlation coefficient: 0.225) (Figure 6D). This result supports the *in vitro* study results, validating the results from NTUH cohorts, and strongly implies that high PlGF levels combined with high Flt-1 expression increase cancer invasion and lead to shorter survival.

## Discussion

In this study we demonstrated that CRC cells express PlGF and Flt-1 have higher invasion/migration ability. PlGF increased the invasion/migration ability of colorectal cancer cells by increasing the phosphorylation of p38 MAPK and upregulating MMP9 expression. Overexpression of PlGF decreased the apoptosis mildly, but did not affect the cell proliferation status. These *in vitro* results had been validated by using two independent CRC cohorts and showed that patients with high PlGF and high Flt-1 expression in CRC tissue had a poorer prognosis. These results revealed that, in addition to previously recognized effects on angiogenesis, PlGF also plays a hitherto unappreciated role in CRC carcinogenesis.

How the PlGF regulates CRC carcinogenesis? PlGF has been shown to increase tumor cell migration in lung cancer, leukemic and melanoma cells [24-26]. It also has been shown that the increased migration of leukemic cells was via the p38/ERK pathway resulting in Rho GTPases activation and caveolae formation [24]. In addition, Loesch et al. demonstrated that the p38gamma MAPK cooperated with transcription factor c-Jun in trans-activating MMP9 which resulted in cell invasion [27]. In line with these findings, our data linked the over-expression of PlGF with the upregulation of MMP9 expression by increasing phosphorylation of p38 MAPK in colorectal cancer cells. In addition, knockdown of p38 MAPK, either by chemical inhibition or siRNA, decreased the expression of MMP9 and the ability of tumor cells to migrate. Furthermore, PlGF expression levels in human CRC tissues correlated well with their MMP9 expression. Taken together, these data suggest that in colorectal cancer cells, PlGF induces tumor cell invasion and migration through upregulation of MMP9 expression by increasing p38 phosphorylation.

Flt-1, one of the well studied receptors of both PlGF and VEGF, plays an important role in regulating vasculogenesis and angiogenesis [28,29]. In addition to angiogenesis, the VEGF-Flt-1 connection also plays an important role in the inflammatory process by activating monocytes/macrophages and inducing their migration [16,17,30,31]. Previously, Xu et al. reported that overexpression of PlGF in HCT116 cells decreased tumor growth, cancer cell invasion and angiogenesis [32]. This result appears to contradict our findings and the clinical observations of others that higher expression of PlGF correlates with poor prognosis [2,6,11-15,33,34]. To elucidate this discrepancy, we checked the PlGF major receptor-Flt-1 status of four different colorectal cancer cell lines, and found that Flt-1 was barely detectable in both HCT116 and HT29 cell lines. In fact, we found exogenous PlGF recombinant protein or stable overexpression of PlGF led to increased invasive ability only in cells with Flt-1 expression. These results suggest PlGF may play different roles in CRC cells depending upon whether the Flt-1 receptor is present, though the role of PlGF in tumor angiogenesis remains controversial [35,36].

Flt-1 protein contains two isoforms, one is the trans-membrane receptor (mFlt-1) and the other is the soluble isoform (sFlt-1). sFlt1 is antiangiogenic as it can function as a decoy that traps the VEGF and PlGF, then prevents binding to VEGFR; whereas mFlt1 is proangiogenic [37,38]. Our results demonstrated that high Flt-1 expression in colorectal cancer cells increased their invasive ability, and is associated with poor prognosis. Recently, Yao et al. also found this similar condition and reported that the role of PlGF in tumorigenesis largely consists of promoting autocrine/paracrine growth of tumor cells expressing a functional Flt-1 rather than stimulation of angiogenesis [39].

## Conclusion

In addition to the well known effect on angiogenesis, PlGF/Flt-1 signaling plays a previously unappreciated important role in colorectal carcinogenesis by increasing the phosphorylation of p38 MAPK, thereby upregulating MMP9 expression; resulting in increasing cellular migration/invasion. Blocking PlGF-Flt-1signaling may be an alternative therapy for treating CRC.

## Competing interests

No conflict of interest to be declared by all authors.

## Authors’ contributions

SCW: study concept and design; acquisition of data; analysis and interpretation of data; drafting of the manuscript; obtained funding; approval of the final version of the manuscript. PNT: study concept and design; statistical analysis; analysis and interpretation of data; drafting of the manuscript; critical revision of the manuscript for important intellectual content. MTW: study concept and design; statistical analysis; analysis and interpretation of data; drafting of the manuscript. ZC: technical and material support; drafting of the manuscript. JMW: samples collection; critical revision of the manuscript for important intellectual content. All authors read and approved the final manuscript.
